# Association of Medicaid Insurance With Survival Among Patients With Small Cell Lung Cancer

**DOI:** 10.1001/jamanetworkopen.2020.3277

**Published:** 2020-04-22

**Authors:** Todd A. Pezzi, David L. Schwartz, Katherine M. W. Pisters, Abdallah S. R. Mohamed, James W. Welsh, Joe Y. Chang, Zhongxing Liao, Saumil J. Gandhi, Lauren A. Byers, Bruce D. Minsky, Clifton D. Fuller, Stephen G. Chun

**Affiliations:** 1 Division of Radiation Oncology, The University of Texas MD Anderson Cancer Center, Houston; 2 Department of Radiation Oncology, University of Tennessee Health Science Center, Memphis; 3 Division of Cancer Medicine Division, The University of Texas MD Anderson Cancer Center, Houston

## Abstract

**Importance:**

Small cell lung cancer (SCLC) is an aggressive neoplasm requiring rapid access to subspecialized multidisciplinary care. For this reason, insurance coverage such as Medicaid may be associated with oncologic outcomes in this disproportionately economically vulnerable population. With Medicaid expansion under the Affordable Care Act, it is important to understand outcomes associated with Medicaid coverage among patients with SCLC.

**Objective:**

To determine the association of Medicaid coverage with survival compared with other insurance statuses.

**Design, Setting, and Participants:**

This cohort study included adult patients with limited-stage (LS) and extensive-stage (ES) SCLC in the US National Cancer Database from 2004 to 2013. Data were analyzed in January 2019.

**Main Outcomes and Measures:**

Patients were analyzed with respect to insurance status. Associations of insurance status with survival were interrogated with univariate analyses, multivariable analyses, and propensity score matching.

**Results:**

A total of 181 784 patients with SCLC (93 131 [51.2%] female; median [interquartile range] age; 67 [60-75] years for patients with LS-SCLC and 68 [60-75] years for patients with ES-SCLC) were identified, of whom 70 247 (38.6%) had LS-SCLC and 109 479 (60.2%) had ES-SCLC. On univariate analyses of patients with LS-SCLC, Medicaid coverage was not associated with a survival advantage compared with being uninsured (hazard ratio, 1.02; 95% CI, 0.96-1.08; *P* = .49). Likewise, on multivariable analyses of patients with ES-SCLC, compared with being uninsured, Medicaid coverage was not associated with a survival advantage (hazard ratio, 1.00; 95% CI, 0.96-1.03; *P* = .78). After propensity score matching, median survival was similar between the uninsured and Medicaid groups both among patients with LS-SCLC (14.4 vs 14.1 months; hazard ratio, 1.05; 95% CI, 0.98-1.12; *P* = .17) and those with ES-SCLC (6.3 vs 6.4 months; hazard ratio, 1.00; 95% CI, 0.96-1.04; *P* = .92).

**Conclusions and Relevance:**

Despite of billions of dollars in annual federal and state spending, Medicaid was not associated with improved survival in patients with SCLC compared with being uninsured in the US National Cancer Database. These findings suggest that there are substantial outcome inequalities for SCLC relevant to the policy debate on the Medicaid expansion under the Affordable Care Act.

## Key Points

**Question:**

Is Medicaid coverage associated with a survival benefit compared with being uninsured among US patients with small cell lung cancer (SCLC)?

**Findings:**

This cohort registry analysis of 181 784 patients with SCLC included in the US National Cancer Database found no association of Medicaid coverage with a survival advantage compared with no insurance. Patients with private insurance, managed care plans, and Medicare had better survival than did Medicaid recipients or uninsured patients even after adjusting for confounding factors.

**Meaning:**

Medicaid coverage was not associated with improved overall survival among patients with SCLC, thus highlighting an opportunity for health care policy intervention in this population.

## Teaser

This cohort study examines the association of Medicaid coverage with survival, compared with other insurance statuses, among patients with small cell lung cancer.

## Introduction

Small cell lung cancer (SCLC) is a highly aggressive neoplasm representing 15% to 30% of lung cancers.^1^ For both limited-stage (LS) and extensive-stage (ES) SCLC, access to multidisciplinary care is crucial to optimize tumor control and improve survival. In LS-SCLC, combined modality therapy with concurrent chemotherapy and thoracic radiation therapy have long been demonstrated to improve survival.^2^ Multidisciplinary care has also been proven to play a crucial role in ES-SCLC where cytotoxic chemotherapy,^3^ thoracic radiotherapy,^4^ and PD-1/PD-L1–directed immunotherapy provide a survival benefit.^5^

Despite the benefits of multidisciplinary care in SCLC, barriers to combined modality therapy in the US, such as government insurance coverage,^6,7^ have been identified. Medicaid is a joint federal- and state-funded program designed to provide health insurance coverage for low-income populations. The Medicaid program currently covers approximately 70 million people in the US, at an annual cost of approximately $600 billion, with increasing enrollment expected because of its expansion under the Affordable Care Act.^8,9,10^ Although Medicaid has been demonstrated to improve access to services such as primary and preventive care,^11^ some lines of evidence suggest that Medicaid may be suboptimal for the management of complex cancers that require specialist care. At least in part as a result of lower reimbursement,^12^ access to specialized outpatient care has been shown to be limited by Medicaid coverage in children and adults.^13,14,15^

Previous analyses^6,7,16,17,18^ have evaluated the association of payer status with outcomes of thoracic cancers. Analyses of patients with lung cancer in the Surveillance, Epidemiology and End Results Program and National Cancer Database (NCDB) have found that Medicaid recipients had inferior survival compared with privately insured or Medicare-insured counterparts.^6,7,16,17,18^ An analysis^12^ of the California Cancer Registry from 1997 to 2014 showed that patients with lung cancer enrolled in Medicaid had no improvements in survival over this period, with survival improvements confined to the privately insured and Medicare populations. Similarly, an analysis^19^ of the New Jersey Medicaid population found particularly poor outcomes in new Medicaid enrollees with cancer after implementation of the Affordable Care Act.

These lines of evidence provide the impetus to examine the value of the Medicaid program for the population of patients with SCLC. Using the NCDB, we evaluated the survival of patients with SCLC from 2004 to 2013 to understand the association of the Medicaid program with outcomes.

## Methods

The NCDB is a joint project of the Commission on Cancer of the American College of Surgeons and the American Cancer Society. Data used in this study were derived from a deidentified NCDB file that is not subject to institutional review board review or a requirement for informed consent, in accordance with 45 CFR §46.

Adult (aged ≥18 years) patients with SCLC diagnosed from 2004 through 2013 were retrieved from the NCDB using the *International Classification of Diseases for Oncology, 3rd Edition*, codes for invasive SCLC (8041/3-8045/3). A total of 202 191 patients were identified. Limited-stage SCLC was differentiated from ES-SCLC using the American Joint Committee on Cancer’s *Cancer Staging Manual* (*Sixth Edition *or* Seventh Edition*) classification for clinical or pathologic evidence of metastatic disease. Cases with missing follow-up data (20 407 patients [10.1%]) and missing M-category data (2058 patients [1.1%]) were excluded from analysis.

### Statistical Analysis

Kaplan-Meier survival curves and stratified log-rank tests were used. Univariate analyses and multivariable analyses (MVA) were conducted using Cox proportional hazard models. Variables that were of known clinical significance and/or statistical significance on Cox proportional hazard models were included for propensity score matching, to minimize bias in an attempt to verify results. The 1-to-1, nearest-neighbor method was used without replacement, with a caliper of 0.2. Balance was assessed using mean standardized differences. Statistical significance was considered at 2-sided *P* < .05. Data analysis was performed using SPSS statistical software version 24 (IBM). Data were analyzed in January 2019.

## Results

There were 181 784 patients with SCLC (93 131 [51.2%] female; median [interquartile range] age; 67 [60-75] years for patients with LS-SCLC and 68 [60-75] years for patients with ES-SCLC) identified in the NCDB from 2004 to 2013 for whom follow-up and survival data were available. After excluding 2058 patients for whom there were no M-category data, it was determined that 70 247 patients (38.6%) had LS-SCLC and 109 479 patients (60.2%) had ES-SCLC. Baseline demographic and clinical characteristics are shown in Table 1. Most patients (94 860 [52.4%]) received care in a comprehensive community cancer program, and most (163 758 [90.1%]) were white. With regard to insurance status, 104 300 patients (57.4%) had Medicare, 12 692 (7.0%) had Medicaid, 51 173 (28.2%) had private or managed plans, 6474 (3.6%) had other insurance, and 7145 (3.9%) had no insurance.

**Table 1.** Baseline Characteristics of Patients With Small Cell Lung Cancer in the US National Cancer Database 2004 to 2013

Characteristic	Patients with small cell lung cancer, No. (%)		
	Limited stage	Extensive stage	Total
Sample size	70 247 (38.6)	109 479 (60.2)	181 784 (100.0)
Facility type			
Community cancer program	10 342 (14.8)	16 757 (15.4)	27 381 (15.1)
Comprehensive community cancer program	37 178 (53.1)	56 672 (51.9)	94 860 (52.4)
Academic or research program	17 675 (25.2)	28 245 (25.9)	46 566 (25.7)
Integrated network cancer program	4719 (6.7)	7296 (6.7)	12 130 (6.7)
Other specified types of cancer programs	93 (0.1)	145 (0.1)	238 (0.1)
Insurance status			
No insurance	2266 (3.2)	4806 (4.4)	7145 (3.9)
Private or managed care	20 055 (28.5)	30 610 (28.0)	51 173 (28.2)
Medicaid	4437 (6.3)	8092 (7.4)	12 692 (7.0)
Medicare	40 984 (58.3)	62 151 (56.8)	104 300 (57.4)
Other	2505 (3.6)	3820 (3.5)	6474 (3.6)
Sex			
Male	31 381 (44.7)	56 352 (51.5)	88 653 (48.8)
Female	38 866 (55.3)	53 127 (48.5)	93 131 (51.2)
Age, median (interquartile range), y	68 (60-75)	67 (60-75)	
Ethnicity			
White	63 081 (89.8)	98 884 (90.3)	163 758 (90.1)
Black	5495 (7.8)	8015 (7.3)	13 702 (7.5)
Other	1133 (1.6)	1706 (1.6)	2894 (1.6)
Unknown	538 (0.8)	874 (0.8)	1430 (0.8)
Charlson-Deyo Comorbidity Index score			
0	40 800 (58.1)	61 366 (56.1)	103 458 (56.9)
1	20 332 (28.9)	32 331 (29.5)	53 176 (29.3)
2	9115 (13.0)	15 782 (14.4)	25 150 (13.8)
T category			
T1	13 341 (19.0)	9925 (9.1)	23 495 (12.9)
T2	18 028 (25.7)	20 643 (18.9)	38 997 (21.5)
T3	7201 (10.3)	10 442 (9.5)	17 788 (9.8)
T4	18 248 (26.0)	37 142 (33.9)	55 665 (30.6)
TX	13 429 (19.1)	31 327 (28.6)	45 839 (25.2)
N category			
N0	13 615 (19.4)	12 652 (11.6)	27 149 (14.9)
N1	6683 (9.5)	7794 (7.1)	14 609 (8.0)
N2	30 563 (43.5)	47 070 (43.0)	78 188 (43.0)
N3	9040 (12.9)	19 711 (18.0)	28 910 (15.9)
NX	10 346 (14.7)	22 252 (20.3)	32 928 (18.1)

For patients with LS-SCLC, associations with overall survival were determined by univariate analyses (eTable 1 in the Supplement) and MVA (Table 2). Medicaid coverage was not associated with survival on univariate analyses (hazard ratio [HR], 1.02; 95% CI, 0.96-1.08; *P* = .49) or MVA (HR, 1.06; 95% CI, 1.00-1.12, *P* = .06) when compared with being uninsured. Factors associated with a statistically significant overall survival benefit on MVA included private or managed care insurance (HR, 0.82; 95% CI, 0.78-0.87; *P* < .001), Medicare insurance (HR, 0.92; 95% CI, 0.88-0.97; *P* = .002), treatment at a noncommunity cancer program (comprehensive community cancer program, HR, 0.93 [95% CI, 0.90-0.95]; academic or research program, HR, 0.85 [95% CI, 0.83-0.87]; integrated network cancer program, HR, 0.91 [95% CI, 0.87-0.94]; other specified types of cancer programs, HR, 0.57 [95% CI, 0.44-0.74]; all *P* < .001), female sex (HR, 0.85; 95% CI, 0.84-0.87; *P* < .001), chemotherapy delivery (HR, 0.62; 95% CI, 0.61-0.64; *P* < .001), and radiation therapy (HR, 0.61; 95% CI, 0.60-0.63, *P* < .001).

**Table 2.** Multivariable Analysis of Factors Associated With Survival Among Patients With Limited-Stage Small Cell Lung Cancer

Variable	HR (95% CI)	*P* value
Facility type		
Community cancer program	1 [Reference]	
Comprehensive community cancer program	0.93 (0.90-0.95)	<.001
Academic or research program	0.85 (0.83-0.87)	<.001
Integrated network cancer program	0.91 (0.87-0.94)	<.001
Other specified types of cancer programs	0.57 (0.44-0.74)	<.001
Insurance status		
No insurance	1 [Reference]	
Private or managed care	0.82 (0.78-0.87)	<.001
Medicaid	1.06 (1.00-1.12)	.06
Medicare	0.92 (0.88-0.97)	.002
Other	0.91 (0.86-0.98)	.01
Sex		
Male	1 [Reference]	
Female	0.85 (0.84-0.87)	<.001
Age (continuous)	1.02 (1.02-1.03)	<.001
Charlson-Deyo Comorbidity Index score		
0	1 [Reference]	
1	1.16 (1.14-1.18)	<.001
2	1.42 (1.39-1.46)	<.001
T category		
T1	1 [Reference]	
T2	1.33 (1.30-1.37)	<.001
T3	1.57 (1.52-1.62)	<.001
T4	1.78 (1.73-1.82)	<.001
TX	1.31 (1.27-1.35)	<.001
N category		
N0	1 [Reference]	
N1	1.31 (1.26-1.35)	<.001
N2	1.62 (1.58-1.66)	<.001
N3	1.86 (1.80-1.92)	<.001
NX	1.62 (1.57-1.68)	<.001
Chemotherapy		
None	1 [Reference]	
Single-agent or multiagent chemotherapy	0.62 (0.61-0.64)	<.001
Chemotherapy contraindicated	1.87 (1.76-2.00)	<.001
Recommended, but not administered	1.08 (1.04-1.13)	<.001
Unknown	0.58 (0.52-0.65)	<.001
Radiation therapy		
Not received	1 [Reference]	
Received	0.61 (0.60-0.63)	<.001
Unknown	0.52 (0.47-0.59)	<.001

Abbreviation: HR, hazard ratio.

Survival analyses were also performed for patients with ES-SCLC to determine factors associated with survival on univariate analyses (eTable 2 in the Supplement) and MVA (Table 3). Compared with being uninsured, Medicaid coverage was not associated with a survival benefit on MVA (HR, 1.00; 95% CI, 0.96-1.03; *P* = .78), whereas private or managed care (HR, 0.86; 95% CI, 0.83-0.89; *P* < .001) and Medicare insurance (HR, 0.94; 95% CI, 0.91-0.97; *P* < .001) were associated with significantly improved survival. Other factors associated with improved survival on MVA included treatment at an academic or research program (HR, 0.93; 95% CI, 0.91-0.95; *P* < .001), treatment at an integrated network cancer program (HR, 0.96; 95% CI, 0.93-0.99; *P* = .004), female sex (HR, 0.87; 95% CI, 0.86-0.88; *P* < .001), chemotherapy delivery (HR, 0.40; 95% CI, 0.39-0.40; *P* < .001), and radiation therapy (HR, 0.78; 95% CI, 0.77-0.79; *P* < .001).

**Table 3.** Multivariable Analysis of Factors Associated With Survival Among Patients With Extensive-Stage Small Cell Lung Cancer

Variable	HR (95% CI)	*P* value
Facility type		
Community cancer program	1 [Reference]	
Comprehensive community cancer program	1.00 (0.98-1.02)	.94
Academic or research program	0.93 (0.91-0.95)	<.001
Integrated network cancer program	0.96 (0.93-0.99)	.004
Other specified types of cancer programs	0.95 (0.80-1.14)	.60
Insurance status		
No insurance	1 [Reference]	
Private or managed care	0.86 (0.83-0.89)	<.001
Medicaid	1.00 (0.96-1.03)	.78
Medicare	0.94 (0.91-0.97)	<.001
Other	0.91 (0.87-0.95)	<.001
Sex		
Male	1 [Reference]	
Female	0.87 (0.86-0.88)	<.001
Age (continuous)	1.01 (1.01-1.01)	<.001
Charlson-Deyo Comorbidity Index score		
0	1 [Reference]	
1	1.17 (1.15-1.18)	<.001
2	1.40 (1.37-1.42)	<.001
T category		
T1	1 [Reference]	
T2	1.15 (1.12-1.18)	<.001
T3	1.18 (1.15-1.22)	<.001
T4	1.26 (1.23-1.29)	<.001
TX	1.21 (1.18-1.24)	<.001
N category		
N0	1 [Reference]	
N1	1.06 (1.03-1.09)	<.001
N2	1.19 (1.16-1.21)	<.001
N3	1.20 (1.17-1.23)	<.001
NX	1.16 (1.13-1.19)	<.001
Chemotherapy		
None	1 [Reference]	
Single-agent or multiagent chemotherapy	0.40 (0.39-0.40)	<.001
Chemotherapy contraindicated	1.63 (1.56-1.69)	<.001
Recommended, but not administered	1.08 (1.06-1.11)	<.001
Unknown	0.48 (0.43-0.54)	<.001
Radiation therapy		
Not received	1 [Reference]	
Received	0.78 (0.77-0.79)	<.001
Unknown	0.54 (0.49-0.60)	<.001

Abbreviation: HR, hazard ratio.

To further adjust for the potentially confounding effect of imbalances in baseline prognostic factors, propensity score matching was performed to further compare survival of patients with SCLC enrolled in Medicaid vs those who were uninsured. The distribution of major prognostic variables of Medicaid recipients and uninsured patients with SCLC after propensity score matching is shown in eTable 3 in the Supplement. A total of 2226 of 2227 uninsured patients with LS-SCLC were matched successfully against 4368 Medicaid recipients with LS-SCLC, and 4748 of 4806 uninsured patients with ES-SCLC were matched successfully against 8092 Medicaid recipients with ES-SCLC. Kaplan-Meier curves of the propensity-matched samples of patients with LS-SCLC (HR, 1.064; 95% CI, 0.996-1.137; *P* = .07) and patients with ES-SCLC (HR, 1.004; 95% CI, 0.963-1.047; *P* = .85) are shown in the Figure. Log-rank estimates showed no significant difference in overall survival between Medicaid recipients and uninsured patients. After fitting an adjusted Cox proportional hazard model of the propensity score–matched sample in patients with LS-SCLC (Table 4), there was no statistically significant difference in overall survival between uninsured patients and those enrolled in Medicaid (median survival, 14.4 vs 14.1 months; HR, 1.05; 95% CI, 0.98-1.12, *P* = .17). Among patients with ES-SCLC, propensity score matching similarly found no statistically significant survival difference between uninsured patients and Medicaid recipients (median survival, 6.3 vs 6.4 months; HR, 1.00; 95% CI, 0.96-1.04, *P* = .92). Factors associated with improved survival were treatment in an academic or research program (HR, 0.82; 95% CI, 0.74-0.91; *P* < .001) or integrated network cancer program (HR, 0.84; 95% CI, 0.72-0.97; *P* = .02) and female sex (HR, 0.81; 95% CI, 0.76-0.87; *P* < .001) (Table 4).

**Figure.**
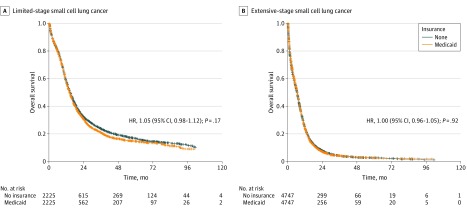
Kaplan-Meier Curves for Overall Survival of Patients With Small Cell Lung Cancer After Propensity Score Matching Graphs show overall survival curves for propensity score–matched cohorts of patients with limited-stage small cell lung cancer (A) and extensive-stage small cell lung cancer (B).

**Table 4.** Cox Multivariable Analysis of Propensity Matched Sample for Patients With Limited-Stage and Extensive-Stage Small Cell Lung Cancer

Variable	HR (95% CI)	*P* value
Patients with limited-stage small cell lung cancer		
Facility type		
Community cancer program	1 [Reference]	
Comprehensive community cancer program	0.93 (0.85-1.02)	.14
Academic or research program	0.82 (0.74-0.91)	<.001
Integrated network cancer program	0.84 (0.72-0.97)	.02
Other specified types of cancer programs	0.58 (0.22-1.55)	.28
Insurance status		
None	1 [Reference]	
Medicaid	1.05 (0.98-1.12)	.17
Sex		
Male	1 [Reference]	
Female	0.81 (0.76-0.87)	<.001
Age, y		
18-50	1 [Reference]	
51-64	1.17 (1.07-1.28)	.001
65-75	1.22 (1.06-1.41)	.01
76-90	2.20 (1.79-2.69)	<.001
Charlson-Deyo Comorbidity Index score		
0	1 [Reference]	
1	1.16 (1.08-1.25)	<.001
2	1.48 (1.32-1.65)	<.001
T category		
T1	1 [Reference]	
T2	1.30 (1.15-1.47)	<.001
T3	1.63 (1.42-1.86)	<.001
T4	1.71 (1.52-1.92)	<.001
TX	1.38 (1.20-1.59)	<.001
N category		
N0	1 [Reference]	
N1	1.32 (1.15-1.52)	<.001
N2	1.50 (1.36-1.66)	<.001
N3	1.71 (1.52-1.92)	<.001
NX	1.65 (1.43-1.90)	<.001
Chemotherapy		
None	1 [Reference]	
Yes	0.66 (0.60-0.73)	<.001
Radiation therapy		
Not received	[Reference]	
Received	0.59 (0.55-0.64)	<.001
Patients with extensive-stage small cell lung cancer		
Facility type		
Community cancer program	1 [Reference]	
Comprehensive community cancer program	1.03 (0.97-1.10)	.31
Academic or research program	0.93 (0.87-0.99)	.03
Integrated network cancer program	0.89 (0.81-0.98)	.02
Other specified types of cancer programs	1.85 (0.77-4.46)	.17
Insurance status		
None	1 [Reference]	
Medicaid	1.00 (0.96-1.04)	.92
Sex		
Male	1 [Reference]	
Female	0.88 (0.85-0.92)	<.001
Age, y		
18-50	1 [Reference]	
51-64	1.05 (0.99-1.11)	.10
65-75	1.05 (0.96-1.16)	.27
76-90	1.02 (0.90-1.17)	.76
Charlson-Deyo Comorbidity Index score		
0	1 [Reference]	
1	1.10 (1.05-1.16)	<.001
2	1.35 (1.26-1.44)	<.001
T category		
T1	1 [Reference]	
T2	1.10 (1.00-1.21)	.04
T3	1.14 (1.03-1.26)	.01
T4	1.20 (1.11-1.31)	<.001
TX	1.25 (1.14-1.37)	<.001
N category		
N0	1 [Reference]	
N1	1.08 (0.98-1.20)	.12
N2	1.16 (1.09-1.25)	<.001
N3	1.19 (1.10-1.28)	<.001
NX	1.10 (1.02-1.19)	.02
Chemotherapy		
None	1 [Reference]	
Yes	0.39 (0.38-0.41)	<.001
Radiation therapy		
Not received	1 [Reference]	
Received	0.77 (0.74-0.80)	<.001

Abbreviation: HR, hazard ratio.

## Discussion

In this analysis, compared with being uninsured, Medicaid coverage was not associated with a survival advantage in patients with SCLC. These findings are directly relevant to the current policy debate on Medicaid expansion under the Affordable Care Act. This study intentionally focused on patients with SCLC because they are often economically disadvantaged, but require timely access to high-quality multidisciplinary care, making them disproportionately vulnerable to poor outcomes.

Although there are patient and tumor factors that could confound these results, we have used propensity score matching to attempt to adjust for inherent biases. This study is unique, to our knowledge, because most prior studies examining SCLC outcomes in Medicaid recipients controlled only for tumor stage without robust adjusted propensity score–matching analyses. Because the NCDB only captures insurance status at the time of initial oncologic treatment, a possible explanation for the lack of apparent differences in survival between the uninsured and Medicaid populations is possible crossover. However, particularly in ES-SCLC, where median survival was approximately 6 months, we think it is unlikely that crossover could explain these results given that it can sometimes take many weeks or even months to process a Medicaid application. Although our group has previously found disparities in treatment depending on insurance status,^6^ the present analysis attempts to control for unequal access to treatment in analyzing survival outcomes.

Disparate outcomes in SCLC can be explained, in part, by multiple patient-related factors, including race/ethnicity, income, comorbidities, education, and insurance status. It is known that low-income individuals experience disparate outcomes in cancer care and that Medicaid recipients tend to present with more-advanced stage disease.^20^ However, the inferior cancer outcomes observed in Medicaid recipients appear to persist even after controlling for cancer stage at presentation. There are a number of factors that may explain the apparent lack of efficacy of Medicaid coverage in SCLC, including higher out-of-pocket drug expenses, limited access to clinical trials, reduced reimbursement, and lengthy processing delays that can be particularly devastating for a cancer with an aggressive histologic profile, such as SCLC. In terms of quality of care for the Medicaid population, a previous meta-analysis^21^ found that uninsured patients were more likely to receive guideline-concordant care than those with private insurance, suggesting that access to health care services, rather than the quality of care, may be associated with inferior outcomes. Indeed, this meta-analysis is consistent with our previous finding^6^ demonstrating that government insurance such as Medicare or Medicaid was uniquely associated with lower rates of radiation delivery in LS-SCLC. Because the Medicaid-to-Medicare national fee index is approximately 70%, the Centers for Disease Control and Prevention estimates that in 2013 only 69% of physicians accepted Medicaid enrollees as new patients.^22^ Although both the problem and the solution are complex, in 2014 the American Society of Clinical Oncology released 9 Medicaid policy recommendations specifically addressing the need to improve access to high-quality care for low-income individuals.^23^

### Limitations

Notable limitations of this study include those common to most population registry studies, in that the accuracy of the analysis depends on the accuracy of the data entry. In addition, these data are retrospective and subject to bias, although we attempted to control for a portion of this bias with propensity matching. It is also possible that treatment of uninsured patients at academic safety-net systems might partially bias these results, even though we also incorporated academic center facility type into the propensity score–matching analysis. There are also multiple other factors not captured in the NCDB that might provide additional insight into our findings, including clinical trial participation, smoking status, tumor biology, radiation fractionation (daily vs twice daily), chemotherapy dosage, and radiation treatment delays and compliance.

## Conclusions

These findings suggest that the Medicaid program is not associated with a survival benefit compared with being uninsured for patients with either LS-SCLC or ES-SCLC. Although the Medicaid program has expanded under the Affordable Care Act in an attempt to improve access to care, additional policy work is needed to improve cancer outcomes for the uninsured and Medicaid populations with SCLC.

## Notes

Supplement. **eTable 1.** Univariate Analysis of Variables Associated With Survival of LS-SCLC in the NCDB **eTable 2.** Univariate Analysis of Variables Associated With Survival of ES-SCLC in the NCDB **eTable 3.** Distribution of Major Prognostic Variables of Medicaid and Uninsured Patients With SCLC After Propensity Score Matching
