# Supplementary material for: Association of Medicaid Insurance With Survival Among Patients With Small Cell Lung Cancer
Source: JAMA Netw Open. 2020 Apr 22;3(4):e203277. doi: 10.1001/jamanetworkopen.2020.3277 (PMC7177199; doi:10.1001/jamanetworkopen.2020.3277)
Supplement: Supplement. — eTable 1. Univariate Analysis of Variables Associated With Survival of LS-SCLC in the NCDB eTable 2. Univariate Analysis of Variables Associated With Survival of ES-SCLC in the NCDB eTable 3. Distribution of Major Prognostic Variables of Medicaid and Uninsured Patients With SCLC After Propensity Score Matching [file jamanetwopen-3-e203277-s001.pdf]

# Supplementary Online Content

Pezzi TA, Schwartz DL, Pisters KMW, et al. Association of Medicaid insurance with survival among patients with small cell lung cancer. *JAMA Netw Open*. 2020;3(4):e203277.
doi:10.1001/jamanetworkopen.2020.3277

**eTable 1.** Univariate Analysis of Variables Associated With Survival of LS-SCLC in the NCDB

**eTable 2.** Univariate Analysis of Variables Associated With Survival of ES-SCLC in the NCDB

**eTable 3**. Distribution of Major Prognostic Variables of Medicaid and Uninsured Patients With SCLC After Propensity Score Matching

This supplementary material has been provided by the authors to give readers additional information about their work.

Supplemental Appendix

eTable 1. Univariate Analysis of Variables Associated With Survival of LS-SCLC in the NCDB

| | P-value | HR | 95% CI |
|---|---|---|---|
| Facility Type | | | |
| Community Cancer Program (Ref) | | | |
| Comprehensive Community Cancer Program | < 0.001 | 0.93 | 0.91-0.95 |
| Academic/Research Program | < 0.001 | 0.84 | 0.82-0.86 |
| Integrated Network Cancer Program | < 0.001 | 0.90 | 0.87-0.93 |
| Other specified types of cancer programs | < 0.001 | 0.57 | 0.44-0.74 |
| | | | |
| Insurance status | | | |
| No Insurance (Ref) | | | |
| Private/Managed Care | < 0.001 | 0.85 | 0.81-0.89 |
| Medicaid | 0.49 | 1.02 | 0.96-1.08 |
| Medicare | < 0.001 | 1.31 | 1.24-1.37 |
| Other | 0.11 | 1.05 | 0.99-1.12 |
| | | | |
| Gender | | | |
| Male (Ref) | | | |
| Female | < 0.001 | 0.86 | 0.84-0.87 |
| | | | |
| Age (continuous) | < 0.001 | 1.03 | 1.03-1.02 |
| | | | |
| Co-morbidity score | | | |
| 0 (Ref) | | | |
| 1 | | | |
| 2 | < 0.001 | 1.21 | 1.19-1.23 |
| | < 0.001 | 1.57 | 1.54-1.61 |
| T-Stage | | | |
| T1 (Ref) | | | |
| T2 | < 0.001 | 1.29 | 1.26-1.33 |
| T3 | < 0.001 | 1.47 | 1.43-1.52 |
| T4 | < 0.001 | 1.71 | 1.66-1.75 |
| TX | < 0.001 | 1.58 | 1.54-1.63 |
| | | | |
| N-Stage | | | |

© 2020 Pezzi TA et al. *JAMA Network Open.*

| | | | |
|---|---|---|---|
| N0 (Ref) | | | |
| N1 | < 0.001 | 1.13 | 1.09-1.17 |
| N2 | < 0.001 | 1.39 | 1.36-1.43 |
| N3 | < 0.001 | 1.55 | 1.51-1.60 |
| NX | < 0.001 | 1.67 | 1.63-1.72 |
| | | | |
| Chemotherapy | | | |
| None (Ref) | | | |
| Single or Multi-agent Chemotherapy | < 0.001 | 0.49 | 0.47-0.50 |
| Chemotherapy contraindicated | < 0.001 | 1.96 | 1.84-2.08 |
| Recommended, but not administered | < 0.001 | 1.13 | 1.08-1.17 |
| Unknown | < 0.001 | 0.46 | 0.41-0.51 |
| | | | |
| Radiation Therapy | | | |
| Not Received (Ref) | | | |
| Received | < 0.001 | 0.50 | 0.49-0.50 |
| Unknown | < 0.001 | 0.44 | 0.40-0.49 |

HR, hazard ratio; CI, confidence interval; Ref, reference level.

© 2020 Pezzi TA et al. *JAMA Network Open.*

eTable 2. Univariate Analysis of Variables Associated With Survival of ES-SCLC in the NCDB

| | P-value | HR | 95% CI |
|---|---|---|---|
| Facility Type | | | |
| Community Cancer Program (Ref) | | | |
| Comprehensive Community Cancer Program | 0.17 | 0.99 | 0.97-1.01 |
| Academic/Research Program | < 0.001 | 0.90 | 0.88-0.92 |
| Integrated Network Cancer Program | 0.002 | 0.96 | 0.93-0.98 |
| Other specified types of cancer programs | 0.348 | 0.92 | 0.77-1.10 |
| | | | |
| Insurance status | | | |
| No Insurance (Ref) | | | |
| Private/Managed Care | < 0.001 | 0.83 | 0.80-0.86 |
| Medicaid | 0.003 | 0.95 | 0.91-0.98 |
| Medicare | < 0.001 | 1.18 | 1.15-1.22 |
| Other | 0.395 | 0.98 | 0.94-1.03 |
| | | | |
| Gender | | | |
| Male (Ref) | | | |
| Female | < 0.001 | 0.88 | 0.87-0.89 |
| | | | |
| Age (continuous) | < 0.001 | 1.02 | 1.02-1.02 |
| | | | |
| Co-morbidity score | | | |
| 0 (Ref) | | | |
| 1 | < 0.001 | 1.22 | 1.20-1.24 |
| 2 | < 0.001 | 1.54 | 1.51-1.57 |
| | | | |
| T-Stage | | | |
| T1 (Ref) | | | |
| T2 | < 0.001 | 1.13 | 1.10-1.15 |
| T3 | < 0.001 | 1.15 | 1.12-1.19 |
| T4 | < 0.001 | 1.23 | 1.20-1.26 |
| TX | < 0.001 | 1.26 | 1.23-1.29 |
| | | | |
| N-Stage | | | |
| N0 (Ref) | | | |
| N1 | 0.764 | 1.00 | 0.97-1.03 |
| N2 | < 0.001 | 1.10 | 1.08-1.12 |

© 2020 Pezzi TA et al. *JAMA Network Open.*

| | | | |
|---|---|---|---|
| N3 | < 0.001 | 1.07 | 1.04-1.09 |
| NX | < 0.001 | 1.19 | 1.16-1.21 |
| | | | |
| Chemotherapy | | | |
| None (Ref) | | | |
| Single or Multi-agent Chemotherapy | < 0.001 | 0.37 | 0.36-0.37 |
| Chemotherapy contraindicated | < 0.001 | 1.79 | 1.72-1.86 |
| Recommended, but not administered | < 0.001 | 1.11 | 1.08-1.14 |
| Unknown | < 0.001 | 0.39 | 0.35-0.43 |
| | | | |
| Radiation Therapy | | | |
| Not Received (Ref) | | | |
| Received | < 0.001 | 0.65 | 0.64-1.66 |
| Unknown | < 0.001 | 0.50 | 0.45-0.55 |

HR, hazard ratio; CI, confidence interval; Ref, reference level.

© 2020 Pezzi TA et al. *JAMA Network Open.*

eTable 3. Distribution of Major Prognostic Variables of Medicaid and Uninsured Patients With SCLC After Propensity Score Matching

| | | Limited-Stage | | Extensive-Stage | |
|---|---|---|---|---|---|
| **Insurance** | | Uninsured | Medicaid | Uninsured | Medicaid |
| **Age** | 18-49 | 383 | 436 | 750 | 730 |
| | | 17.2% | 19.6% | 15.8% | 15.4% |
| | 50-64 | 1648 | 1510 | 3596 | 3429 |
| | | 74.0% | 67.8% | 75.7% | 72.2% |
| | 65-75 | 137 | 203 | 307 | 414 |
| | | 6.2% | 9.1% | 6.5% | 8.7% |
| | 76-90 | 58 | 77 | 95 | 175 |
| | | 2.6% | 3.5% | 2.0% | 3.7% |
| Total | | 2226 | 2226 | 4748 | 4748 |
| | | 100.0% | 100.0% | 100.0% | 100.0% |
| | | | | | |
| Sex | Male | 1013 | 1069 | 2531 | 2507 |
| | | 45.5% | 48.0% | 53.3% | 52.8% |
| | Female | 1213 | 1157 | 2217 | 2241 |
| | | 54.5% | 52.0% | 46.7% | 47.2% |
| Total | | 2226 | 2226 | 4748 | 4748 |
| | | 100.0% | 100.0% | 100.0% | 100.0% |
| | | | | | |
| Facility Type | Community Cancer Program | 329 | 361 | 694 | 782 |
| | | 14.8% | 16.2% | 14.6% | 16.5% |
| | Comprehensive Community Cancer Program | 1080 | 1025 | 2310 | 2035 |
| | | 48.5% | 46.0% | 48.7% | 42.9% |
| | Academic/Research Program | 657 | 661 | 1435 | 1527 |
| | | 29.5% | 29.7% | 30.2% | 32.2% |
| | Integrated Network Cancer Program | 159 | 178 | 306 | 402 |
| | | 7.1% | 8.0% | 6.4% | 8.5% |
| | Other specified types of cancer programs | 1 | 1 | 3 | 2 |
| | | 0.0% | 0.0% | 0.1% | 0.0% |
| Total | | 2226 | 2226 | 4748 | 4748 |
| | | 100.0% | 100.0% | 100.0% | 100.0% |
| | | | | | |
| Charlson/Deyo Score | 0 | 1399 | 1263 | 2988 | 2642 |
| | | 62.8% | 56.7% | 62.9% | 55.6% |
| | 1 | 643 | 696 | 1302 | 1403 |

© 2020 Pezzi TA et al. *JAMA Network Open.*

| | | | | | |
|---|---|---|---|---|---|
| | | 28.9% | 31.3% | 27.4% | 29.5% |
| | 2 | 184 | 267 | 458 | 703 |
| | | 8.3% | 12.0% | 9.6% | 14.8% |
| Total | | 2226 | 2226 | 4748 | 4748 |
| | | 100.0% | 100.0% | 100.0% | 100.0% |
| | | | | | |
| T Stage | T1 | 282 | 268 | 359 | 373 |
| | | 12.7% | 12.0% | 7.6% | 7.9% |
| | T2 | 484 | 588 | 782 | 991 |
| | | 21.7% | 26.4% | 16.5% | 20.9% |
| | T3 | 307 | 324 | 489 | 640 |
| | | 13.8% | 14.6% | 10.3% | 13.5% |
| | T4 | 779 | 645 | 1897 | 1495 |
| | | 35.0% | 29.0% | 40.0% | 31.5% |
| | TX | 374 | 401 | 1221 | 1249 |
| | | 16.8% | 18.0% | 25.7% | 26.3% |
| Total | | 2226 | 2226 | 4748 | 4748 |
| | | 100.0% | 100.0% | 100.0% | 100.0% |
| | | | | | |
| N Stage | N0 | 357 | 433 | 537 | 699 |
| | | 16.0% | 19.5% | 11.3% | 14.7% |
| | N1 | 170 | 246 | 295 | 389 |
| | | 7.6% | 11.1% | 6.2% | 8.2% |
| | N2 | 1024 | 821 | 1980 | 1747 |
| | | 46.0% | 36.9% | 41.7% | 36.8% |
| | N3 | 376 | 435 | 1014 | 1074 |
| | | 16.9% | 19.5% | 21.4% | 22.6% |
| | Nx | 299 | 291 | 922 | 839 |
| | | 13.4% | 13.1% | 19.4% | 17.7% |
| Total | | 2226 | 2226 | 4748 | 4748 |
| | | 100.0% | 100.0% | 100.0% | 100.0% |
| | | | | | |
| Radiation Delivery | No | 760 | 808 | 2700 | 2800 |
| | | 34.1% | 36.3% | 56.9% | 59.0% |
| | Yes | 1454 | 1403 | 2032 | 1925 |
| | | 65.3% | 63.0% | 42.8% | 40.5% |
| | No Data | 12 | 15 | 16 | 23 |
| | | 0.5% | 0.7% | 0.3% | 0.5% |
| Total | | 2226 | 2226 | 4748 | 4748 |

© 2020 Pezzi TA et al. *JAMA Network Open.*

| | | | | | |
|---|---|---|---|---|---|
| | | 100.0% | 100.0% | 100.0% | 100.0% |
| | | | | | |
| Chemotherapy Delivery | not received | 408 | 420 | 1435 | 1474 |
| | | 18.3% | 18.9% | 30.2% | 31.0% |
| | received | 1804 | 1796 | 3304 | 3257 |
| | | 81.0% | 80.7% | 69.6% | 68.6% |
| | unknown | 14 | 10 | 9 | 17 |
| | | 0.6% | 0.4% | 0.2% | 0.4% |
| Total | | 2226 | 2226 | 4748 | 4748 |
| | | 100.0% | 100.0% | 100.0% | 100.0% |

© 2020 Pezzi TA et al. *JAMA Network Open.*
